# Synchronization Analysis of Master-Slave Probabilistic Boolean Networks

**DOI:** 10.1038/srep13437

**Published:** 2015-08-28

**Authors:** Jianquan Lu, Jie Zhong, Lulu Li, Daniel W. C. Ho, Jinde Cao

**Affiliations:** 1 Department of Mathematics, Southeast University, Nanjing 210096, China; 2 School of Automation, Southeast University, Nanjing, 210096, China; 3 School of Mathematics, Hefei University of Technology, Hefei, 230009, China; 4 Department of Mathematics, City University of Hong Kong, Kowloon, Hong Kong; 5 Department of Mathematics, Faculty of Science, King Abdulaziz University, Jeddah 21589, Saudi Arabia

## Abstract

In this paper, we analyze the synchronization problem of master-slave probabilistic Boolean networks (PBNs). The master Boolean network (BN) is a deterministic BN, while the slave BN is determined by a series of possible logical functions with certain probability at each discrete time point. In this paper, we firstly define the synchronization of master-slave PBNs with probability one, and then we investigate synchronization with probability one. By resorting to new approach called semi-tensor product (STP), the master-slave PBNs are expressed in equivalent algebraic forms. Based on the algebraic form, some necessary and sufficient criteria are derived to guarantee synchronization with probability one. Further, we study the synchronization of master-slave PBNs in probability. Synchronization in probability implies that for any initial states, the master BN can be synchronized by the slave BN with certain probability, while synchronization with probability one implies that master BN can be synchronized by the slave BN with probability one. Based on the equivalent algebraic form, some efficient conditions are derived to guarantee synchronization in probability. Finally, several numerical examples are presented to show the effectiveness of the main results.

Recently, researches on the behavior and close relationships of all the RNAs, DNAs, proteins, and cells in a genetic regulatory network have been a new hot topic^1^,^2^. Boolean networks (BNs) were originally introduced to model large-scale genetic regulatory networks^3^,^4^,^5^, and then they have become a powerful and appropriate tool to model long-term behavior of genes. On one hand, BNs can be a convenient model to describe lots of phenomena whose describing variables display only two operation values (active/inactive, on/off, …). For example, each gene in a cell behaves just like a switch, switching either on “active” or on “inactive”, which can also be expressed by 1 and 0. Meanwhile, each gene is activated or inhibited by a series of Boolean functions. Great attention has been paid to the study of BNs, such as investigation of topological structure of BNs, including the fixed point, cycles, attractors and transient time^6^,^7^,^8^. On the other hand, the algebraic state representation for BNs, developed by Cheng and co-authors, allows to convert BNs into the framework of linear state-space models^9^,^10^,^11^. Cheng and his group develop a new matrix product, called semi-tenor product (STP) of matrices, which presents a new way to multiply two matrices with arbitrary dimensions^9^. By resorting to STP, a Boolean function can be converted into an algebraic form, and then a BN (Boolean network) can be expressed as a discrete algebraic dynamic^10^. This original set-up opens new perspectives on systematical analysis of many problems about BNs. And, indeed, using this approach, many problems concerning BNs like stabilization^12^, controllability^13^,^14^,^15^,^16^,^17^, observability^18^, optimal control^19^, and synchronization^20^,^21^, just quote a few, have been widely investigated.

It should be noted that one main drawback of the algebraic state expression of BNs is its computational complexity. The algebraic state representation converts a BN with *n* state-variables into a state-space of size 2^*n*^. Thus, any algorithm based on this approach has an exponential time-complexity. Moreover, many problems like determining fixed points and observability of Boolean control networks have already been proved to be NP-hard. Hence, the computational complexity is intrinsic and also independent of the models adopted to describe BNs.

It is a curious phenomenon of some real-world systems that they can evolve in perfect synchronization. Synchronization is an important property, which makes two coupled systems oscillate in typical collective behavior. In recent years, synchronization problem of dynamic systems have drawn great attention, sucn as synchronization of complex networks^22^,^23^,^24^, consensus in multi-agent systems^25^,^26^, synchronization of Kauffman networks^27^, cooperation of networks^28^,^29^,^30^,^31^ and so on. Since BNs can provide general features of living organism, and well illustrate genetic regulatory networks, the synchronization problem has been extended to BNs. The researches on synchronization of BNs can provide lots of useful information on the evolution of biological systems whose corresponding subsystem influences with each other. For example, investigation on synchronized BNs is beneficial to better understand synchronization between two coupled lasers^32^. Hence, studying the synchronization problem of BN is of both theoretical and practical importance. In the past few years, Some necessary and sufficient criteria of complete synchronization for two deterministic BNs has been obtained^33^, then Li *et al.* generalized the synchronization problem of BNs with time delays^34^. In^35^, Li studied synchronization of coupled large-scale BNs. In^36^, Zhong *et al.* have investigated synchronization of master-slave BNs with impulsive effects.

In^9^,^13^,^37^, the target state of nodes in BNs is predicted by deterministic Boolean functions. Deterministic BNs always follow a static transition mechanism supervised by binary logical functions, and ultimately reach a limit set, from which the system cannot move. However, the stochastic feature of genetic regulation and micro array data used to infer the structure of networks may have errors because of external noise in the complex measurement processes. Hence, the stochastic factor is an important feature, and BNs with stochastic factor is more practical and favorable to such situations, resulting in the development of probabilistic Boolean networks (PBNs). In^38^, Shmulevich *et al.* firstly proposed PBNs model, which deals with the problem of uncertainty. A PBN can be regarded as a collection of BNs, in which the state of each node chooses its transition rule according to some probabilistic rules at discrete time point. And the transition rule for updating each node is randomly chosen among several possible rules with a given probability distribution. Hence, a PBN allows the model to have more flexibility, which is the basic idea of PBNs.

Recently, PBNs have been widely applied to infer functional connectivity between brain regions and to investigate the connectivity abnormality in Parkinson’s Disease^39^. Some fundamental and interesting results on PBNs have been obtained, such as optimal control problem in context-sensitive PBNs^40^, controllability of PBNs with forbidden states^17^, steady-state probability distribution of PBNs^41^. Due to its rule-based and uncertainties properties, PBNs seem more practical to model genetic regulatory networks than usual deterministic BNs. And phenomenon of coupling is very common in real world systems. Hence, it is meaningful and challenging to study the synchronization problem of PBNs, and there has been no result investigating on synchronization of PBNs, to the best our knowledge. Thus, motivated by the above discussions, in this paper, we aim to investigate the synchronization problem of PBNs coupled in the master-slave configuration, in which the master BN is a deterministic BN while the slave BN is a PBN. In this paper, we firstly investigate synchronization of master-slave PBNs with probability one, then investigate synchronization in probability. New approaches based on STP are proposed to derive necessary and sufficient conditions for synchronization.

Notation: The following standard notations will be used in this paper. Throughout this paper, 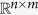 denotes the set of real matrices of order *n* × *m*, and 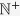 denotes the positive integers. **1**_*n*_ denotes the *n*-dimensional column vector with all entries being 1, and *I*_*k*_ is the identity matrix of order *k*. 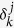 is the *j*-th column of identity matrix *I*_*k*_, and Δ_*k*_ denotes the set of all *k* columns of *I*_*k*_. In particular, when *k* = 2, we use 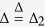. Let Col_*j*_(*A*) (Row_*j*_) be the *j*-th column (*j*-th row) of matrix *A*, and Col(*A*) (Row(*A*)) be the set of columns (rows) of matrix *A*. A *k* × *p* matrix A is called a logical matrix if 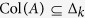, and the set of all *k* × *p* logical matrices is denoted by 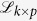.

## Preliminaries

Given two integers 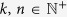, with *k* ≤ *n*, we use [*k*, *n*] to denote the set of integers {*k*, *k* + 1, …, *n*}. A *k* × *p* logical matrix 
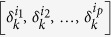
 can be simply written as *δ*_*k*_[*i*_1_, *i*_2_, …, *i*_*p*_], for suitable indices 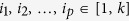. We consider Boolean vectors, taking values in 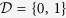 with usual operations (sum +, product ·, and negation ¬). A *k* × *p* matrix A is called a Boolean matrix if *A*_*ij*_ ∈ 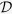 for each *i* ∈ [1, *k*] and *j* ∈ [1, *p*]. Let ⊗ denotes the Kronecker product of matrices.

Firstly, we introduce a bijective correspondence between Boolean vectors *X* ∈ 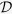 and vectors *x* ∈ Δ, which is defined by the relationship:


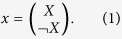


Then, we introduce semi-tensor product (STP) “
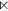
” between matrices (and in particular, vectors) as follows^10^: given two matrices, *L*_1_ ∈ 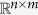 , *L*_2_ ∈ 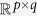, we set


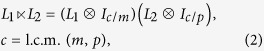


where l.c.m.(*m*, *p*) denotes the least common multiple of *m* and *p*.

As we can see, STP of matrices is an extension of standard matrix product, by this meaning if *m* = *p*, then we can get *L*_1_ 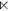 *L*_2_ = *L*_1_ *L*_2_. Note that if *x*_1_ ∈ Δ_*n*1_ and *x*_2_ ∈ Δ_*n*2_, then *x*_1_ 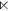 *x*_2_ ∈ Δ_*n*1*n*2_. Throughout this paper, we sometimes just omit “
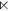
” for convenience. By resorting to STP, we can present a bijective correspondence between 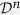 and Δ_2*n*_. It can be obtained in following way: given 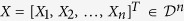, where “*T* ” represents the transpose, we set


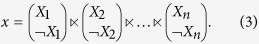


Thus, we can obtain that


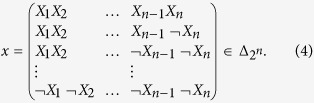


**Example 1** *Consider two matrices* 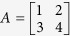, 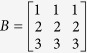. *Then, according to* Eq. (2), *we can obtain the STP of matrices A and B as follows*:


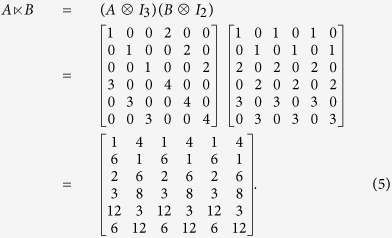


**Definition 1** *An mn* × *mn matrix W*_[*m,n*]_ *is called a swap matrix, if it is constructed in following way: label its columns by* (11, 12, …, 1*n*, …, *m*1, *m*2, …, *mn*) *and similarly label its rows by* (11, 21, …, *m*1, …, 1*n*, 2*n*, …, *mn*). *Then its element in the position* ((*I*, *J*), (*i*, *j*)) *is assigned as*


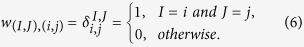


*If σ*_1_ ∈ Δ_*m*_ *and σ*_2_ ∈ Δ_*n*_, *then σ*_1_ 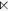 *σ*_2_ = *W*_[*m*,*n*]_(*σ*_2_ 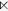 *σ*_1_). *If m* = *n*, *we denote W*_[*m*,*n*]_ *by W*_[*n*]_ *for convenience*.

**Example 2** *According to Definition* 1, *we can construct the swap matrix W*_[3,2]_ *and obtain the matrix W*_[3,2]_ *as following*:


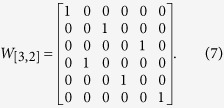


By resorting to STP and the bijective correspondence between 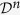 and Δ_2*n*_, we can acquire an algebraic representation of logical functions. To do so, we have to identify the Boolean vectors 1 and 0 with the vectors 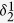 and 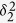. That is to say, we consider a Boolean variable *X* ∈ 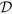 as a vector *x* ∈ Δ, thus a Boolean function of *n* variables 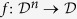 is equivalent with a map 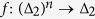. Then, using STP, we can simply express a series of Boolean variables and obtain its equivalent algebraic form of a logical function.

**Lemma 1**^9^ *Let* 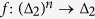 *be a Boolean function. Then there exists a unique matrix* 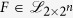 *such that* 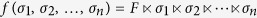, *for every* 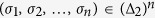. *F is called the structure matrix of the logical function f*.

**Example 3** *Consider the following two logical functions* 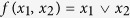 *and* 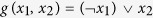. *Then, according to Lemma 1 and the Truth* Table 1, *we can obtain its corresponding structure matrices M*_*f*_ *and M*_*g*_ *satisfying*:


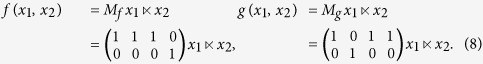


**Lemma 2**^9^

(a) *If σ* ∈ Δ_*n*_, *then* 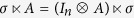 *for every A*.

(b) *If σ* ∈ Δ_2*n*_, *then σ* 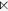 *σ* = Φ_*n*_*σ*, *where  *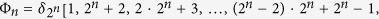 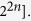


(c) *The dummy matrix is defined as* 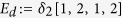. *Then for any two logical variables u*, *v*, *we have E*_*d*_*uv* = *v*, *or E*_*d*_*W*_[2]_*uv* = *u*.

(d) *Let X* ∈ Δ_*m*_ *and Y* ∈ Δ_*n*_ *be two arbitrary columns. Then, according to the definition of swap matrix, we have* 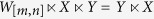, 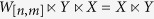.

## Results and Methods

### Matrix expression of master-slave probabilistic Boolean networks (PBNs)

Recall that two BNs coupled in master-slave configuration, and each network has *n* nodes, which can be described as:


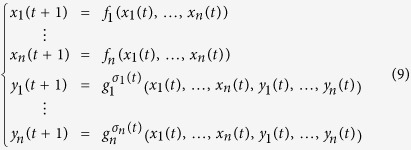


where *x*_*i*_ is the *i*-th node of master BN, and *y*_*i*_ is the *j*-th node of slave BN, respectively. 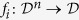, *i* ∈ [1, *n*], 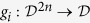, *i* ∈ [1, *n*] are logical functions; 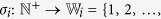 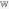
_i_} can be regarded as switching signals; t = 0, 1, 2, …, and here we simply denote 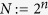. We simply denote 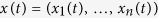 and 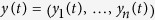 to be the states of the master BN and the slave BN at time instant *t*, respectively. Moreover, we can observe that the state evolution of the master-slave BNs depends on the following initial states: 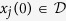, 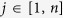 and 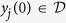, 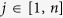.

The master-slave BNs (9) becomes a master-salve PBNs if the probability of *g*_*i*_ being 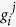 is 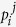, denoted as 
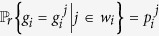
, 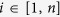, 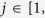 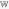
_i_]. That is 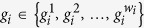 and 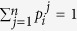, 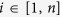. In this section, we assume that the slave PBN is independent, that is *g*_1_, *g*_2_, …, *g*_*n*_ are independent from each other, i.e. 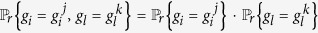.

Using the matrix 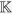 to denote the index set of possible models^38^,


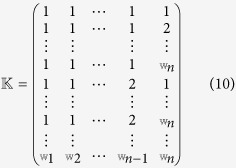


where 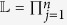

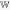
_j_. Thus, 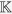 is a 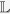 × *n* matrix.

**Remark 1** *If there are some identical switching signals, assuming that* 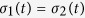 *and* 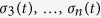 *are pairwise distinct, then we can denote* 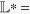
* *
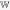
_1_ 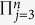

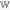
_j_. *Hence, in the following sequel, we assume that the switching signals* 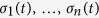 *are pairwise distinct*.

Each row of matrix 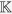 represents a possible network with probability 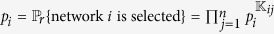, where 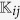 is the *ij*-th entry in matrix 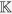. Now define 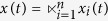, and 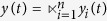, which is a bijective mapping pointed by D. Cheng^9^,^10^. For each logical functions 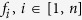, we can find its corresponding structure matrix *F*_*i*_. Thus, using Lemma 1, for the master logical functions, we can obtain its algebraic form:


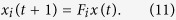


Multiplying Eq. (11) yields 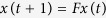, where 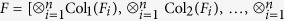 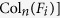.

Then, for each logical functions 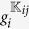, 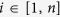, we can find its structure matrix 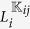. Thus, using Lemma 1, for the slave logical functions, we have


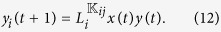


Multiplying Eq. (12) yields that 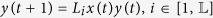, where 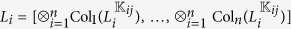.

Thus, for master-slave PBNs (9), we obtain the following equivalent algebraic expression:


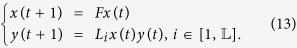


In fact, the master-slave PBNs (9) can be regarded as a whole system. Let 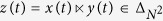 be the state of the whole system. Then for the master-slave PBNs (9), we can obtain following dynamics of the whole system:


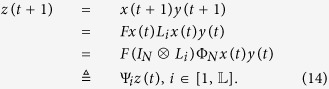


Hence, the overall expected value of *z*(*t* + 1) satisfies:


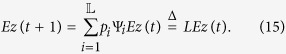


**Remark 2** *According to* Eq. (14), *we know that the state z*(*t* + 1) *is updated by the logical function* Ψ_*i*_ *with a certain probability, i.e. p*_*i*_. *And actually*, *z*(*t* + 1) *has* 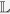 *number of choices to update its states. Unlike deterministic BNs, PBNs do not have accurate state evolutional process, and all the possible state evolutional processes exist with some certain probabilities*.

**Remark 3** *According to* Eq. (14) *and* Eq. (15), *we can obtain that the pq-th entry of matrix L*_*i*_ *is equal to* 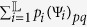, *i.e.* 
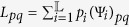
. *Since* 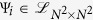, *then we can obtain the following equation:* 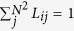, *which means the sum of column entries is unitary*.

**Remark 4** *According to* Eq. (15), *we can observe that if the master BN is a PBN and the slave BN is a deterministic BN, we can still obtain an algebraic equation similar to* Eq. (15). *However, due to the coupling property between master BN and slave BN (slave BN is also affected by master BN), the slave BN is also a PBN. Thus, in order to investigate synchronization for this kind of system, we only need to check whether z*(*t*) (*state of the whole system*) *can reach the set of synchronized states with probability one. Hence, similar methods for synchronization of deterministic BNs can be used to investigate synchronization of these systems*.

**Example 4** *Consider the following master-slave PBNs:*


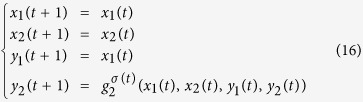


*where the switching signal on logical function g*_2_ *is* 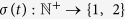 *and*


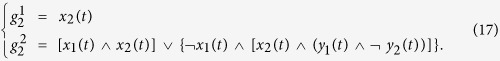


*Here, the probabilities of g*_2_ *being* 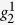 *and* 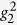 *are* 
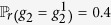
 *and* 
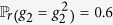
. *Denote* 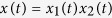 *and* 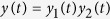. *By resorting to STP and Lemma* 1, *we can obtain its equivalent algebraic form as follows:*


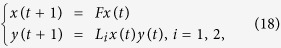


*where the probabilities of L*_*i*_ *being L*_1_ *and L*_2_ *are* 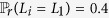 *and* 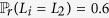, *and*


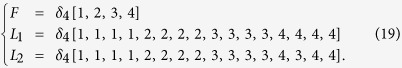


*Further, denote* 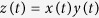, *we can obtain the whole system as follows:* 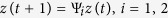, *where*


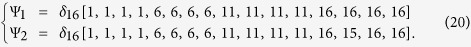


**Figure 1:**
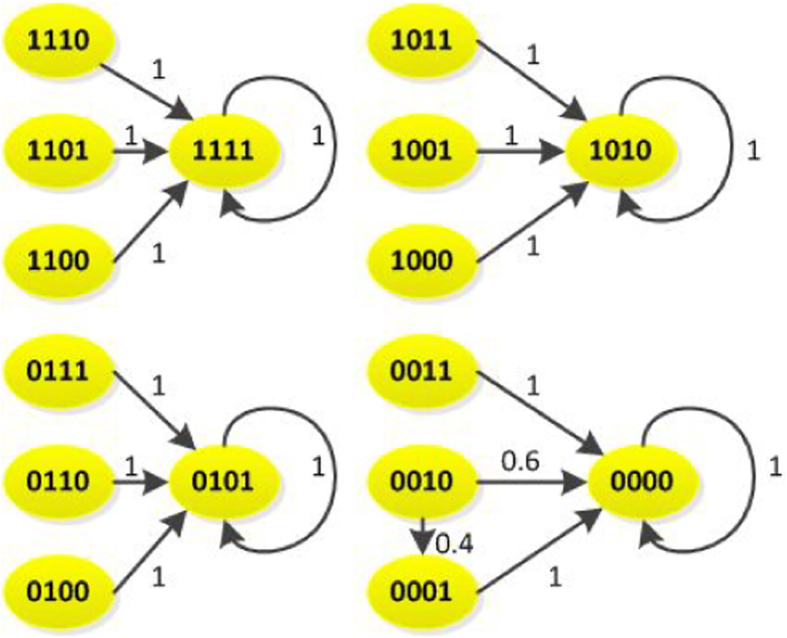
State transition digraph of system (16), where the positive number beside each arrow is the probability under which state transfers to its next state.

*The state transition digraph of system* (16) *is shown in* Fig. 1. *Hence, we can obtain that the overall expected value of z*(*t* + 1) *satisfies:*
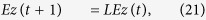
*where*


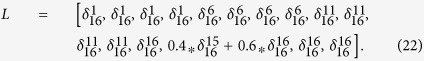


### Synchronization of master-slave PBNs with probability one

In the following sebsection, we firstly define the definition of synchronization of the master-slave PBNs (9) with probability one as follows.

**Definition 2** *Consider the master-slave PBNs* (9). *System* (9) *is said to be synchronized with probability one if for any initial state* 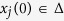, 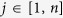 *and* 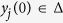, 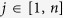, *there exists a positive integer k, such that t* ≥ *k satisfies*


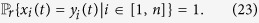


**Remark 5** *If the master-slave PBNs* (9) *can be synchronized with probability one, then there must exist an integer k such that for t* ≥ *k*, 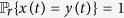. *By this meaning, the slave BN has only one deterministic trajectory after finite steps, which is exactly the same as the trajectory of master BN, i.e. x*(*t*) = *y*(*t*) for *t* ≥ *k*. *Denote* 
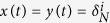
, *according to* 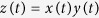, *we have* 
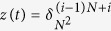
. *Thus, let* 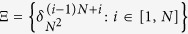 *be the set of synchronized states about z*(*t*). *Let* 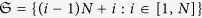 *be the index set of* Ξ.

**Remark 6** *In*^33^,^34^, *Li et al. have investigated the complete synchronization of BNs coupled in drive-response configuration. In those models, the drive BN and response BN are both deterministic BN, which implies that the trajectory of drive BN will coincide with that of response BN after finite steps. Since the stochastic factor is an important feature in real world, BNs with stochastic factor is more practical and favorable. Here, we consider that the master BN is a deterministic BN, while the slave BN is a probabilistic BN. Due to the fact that the master BN is a deterministic BN which means there will be only one trajectory, the slave BN must have only one trajectory coinciding with master BN after finite steps. Thus, the main difference between synchronization with probability one and general synchronization is that there will be some possible trajectories at the beginning of a period time but only one deterministic trajectory after finite steps*.

According to Eq. (9), we observe that the master BN is a deterministic BN. Thus, the trajectory will enter into a cycle after finite steps starting from any state. Let 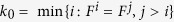 be the transient period of system and *T* > 0 be the smallest positive number satisfying 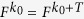. Thus, we can obtain the following proposition.

**Proposition 1** *Starting from any state, the trajectory of master BN* (9) *will enter into a cycle after k*_0_ *steps*.

**Example 5** *Consider the following master BN with* 3 *nodes:*


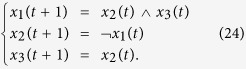


**Figure 2:**
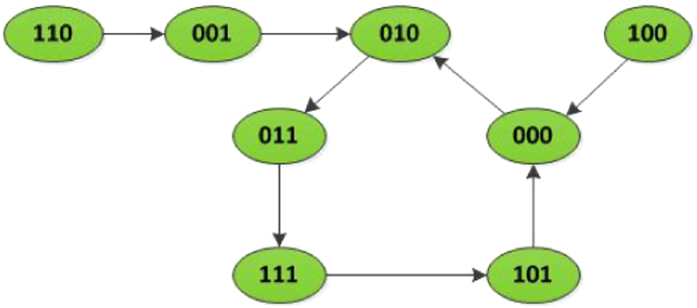
The dynamic graph of system (24).

*Denote* 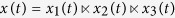, *it is easy to calculate that* 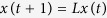, *where L follows immediately as L* = *δ*_8_[3, 7, 8, 8, 1, 5, 6, 6]. *Thus, it is easy to check that k*_0_ = 2 *and L*^2^ = *L*^7^, *i.e. T* = 5, *which implies that the trajectory of BN will enter a cycle after* 2 *steps. The dynamic graph of system* (24) *is shown in* Fig. 2, *from which we can see that each state will enter a cycle with length* 5 *after* 2 *step*.

Based on Proposition 1, we can obtain the following necessary and sufficient condition for synchronization of master-slave PBNs (9) with probability one.

**Theorem 1** *Consider the master-slave PBNs* (9). *System* (9) *can be synchronized with probability one if and only if the following conditions hold:*

• *For* 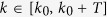,


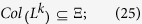


•  


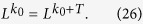


**Proof**. According to Eq. (15), we can obtain that


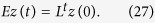


(Necessity) If the master-slave PBNs (9) can be synchronized with probability one, then there must exist an integer *k*, such that for *t* ≥ *k* satisfying 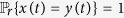. Since the trajectory of master BN will enter into a cycle after *k*_0_ iterations, we only need to consider whether the limit set of slave BN can be coincided with that of master BN or not. For any initial state *x*(0), based on Proposition 1, the trajectory of master BN will reach a cycle: 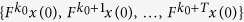. Denote 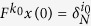, 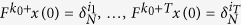. Since 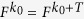, we have 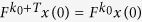, i.e. *i*_*T*_ = *i*_0_. Since the master-slave PBNs (9) can be synchronized with probability one from any initial states *x*(0), *y*(0), we can obtain that the trajectory of slave BN also reach the same cycle: 
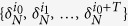
. By this meaning, we have 
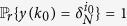
, 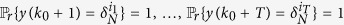. Thus, it is equivalent to


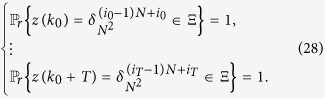


According to Eq. (27), this implies that 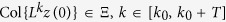. Since the initial state *z*(0) is arbitrary, we can derive that 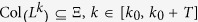. As 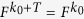, for any initial states *x*(0), *y*(0), we have 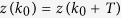 which implies that 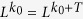. The necessity is proved.

(Sufficiency) Assuming that conditions (25) and (26) hold, we prove that under these conditions the master BN can be synchronized by the slave BN with probability one. Suppose that 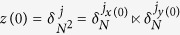, 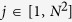, 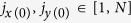. If (25) holds, after *k*_0_ steps, we have 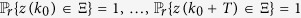. Since the set Ξ is synchronized set, one has


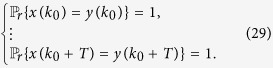


If (26) holds, we obtain that 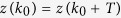, which means that 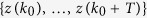 is a cycle. By this meaning, the trajectory of system (14) enter into a cycle. This together with (29) yields that the master BN can be synchronized by the slave BN with probability one, as the index *j* is arbitrary. This completes the proof.

**Remark 7** *According to Theorem* 1, *we observe that condition* (25) *guarantees that the master BN can be synchronized by slave BN for states in limit set with probability one. And condition* (26) *guarantees that the slave BN has the same cycles or fixed points with probability one after k*_0_ *steps. Thus, condition* (26) *is a necessary condition to guarantee synchronization. Even for some systems satisfying condition* (25), *it can not reach synchronization*.

**Remark 8** *According to Proposition* 1, *we can conclude that the trajectory of master BN will enter into a cycle after k*_0_ *steps. To investigate the synchronization with probability one, we only need to consider the following time sequence k*_0_, *k*_0_ + 1, …, *k*_0_ + *T*, *because the matrix F satisfies* 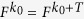. *Since the set* Ξ *is the set of synchronized states, condition* (25) *implies that the slave BN can reach synchronization with probability one at time sequence k*_0_, *k*_0_ + 1, …, *k*_0_ + *T*, *but can not guarantee synchronization after time k*_0_ + *T*. *However, due to the fact that* 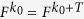, *the slave BN also need to guarantee a periodic trajectory with the same length as the trajectory of master BN if the slave BN wants to reach synchronization. Thus, condition* (26) *guarantees that the periodic trajectory of slave BN coincides with that of master BN*.

According to Theorem 1, we can easily obtain following corollary to check whether a given master-slave PBN can be synchronized with probability one or not.

**Corollary 1** *Consider the master-slave PBNs* (9). *System* (9) *can be synchronized with probability one if and only if the following conditions hold:*

• *For* 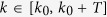, 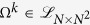, *where matrix* Ω^*k*^ *is the matrix obtained from L*^*k*^ *by deleting the rows with index* 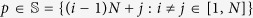;
• 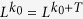.

**Theorem 2** *Consider the master-slave PBNs* (9). *The master-slave PBNs can be synchronized with probability one, if following two conditions hold:*

- (1) *there exists a positive number* 0 < *k* ≤ *k*_0_ + *T*, *such that* 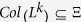;
- (2) *Col*_*j*_(*L*) ∈ Ξ, 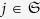.

**Proof**. For any initial states *x*(0), *y*(0), according to Eq. (15) and after *k* iterations, we have 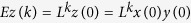. Suppose that condition (1) holds, then we have *Ez*(*k*) ∈ Ξ, which implies that 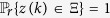. Then for the next step, we have 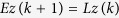. The facts that *z*(*k*) ∈ Ξ and condition (2) holds means that *Ez*(*k* + 1) ∈ Ξ, which further implies that 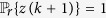. Thus using mathematical iteration, we obtain that 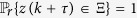, 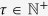. By this meaning, for any initial states *x*(0), *y*(0), we have 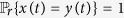, *t* ≥ *k*. Thus, it implies that the master-slave PBNs (9) can be synchronized with probability one.

**Corollary 2** *Consider the master-slave PBNs* (9). *The master-slave PBNs can be synchronized with probability one, if following two conditions hold:*

- (1) *there exists a positive number* 0 < *k* ≤ *k*_0_ + *T*, *such that* 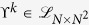, *where matrix* ϒ^*k*^ *is the matrix obtained from L*^*k*^ *by deleting the rows with index* 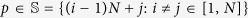;
- (2) 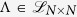, *where matrix* Λ *is the matrix obtained from L by deleting the column and rows with index* 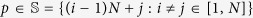.

### Synchronization of master-slave PBNs (9) in probability

In the above section, we have investigated synchronization of master-slave PBNs (9) with probability one. Since the master BN is a deterministic BN, synchronization with probability one implies that the slave BN has deterministic trajectories coinciding with trajectories of master BN after finite steps. As we can see, this condition is relative strict in some real-world systems. If the slave BN has some trajectories coinciding with trajectories of master BN with some certain probability, what happens? Thus in following section, we will investigate synchronization of master-slave PBNs (9) in probability, which implies that the master BN can be synchronized by the slave BN with some certain probability. Now, we firstly define the definition of synchronization in probability as follows.

**Definition 3** *Consider the master-slave PBNs* (9). *System* (9) *is said to be synchronized in probability if for any initial state* 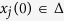, 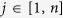 *and* 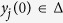, 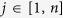, *there exists a positive integer k, such that t* ≥ *k satisfies*


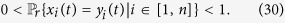


**Remark 9** *In Definition* 2, *we have presented the definition of synchronization with probability one. Since the master BN is a deterministic BN, under this definition of synchronization, the slave BN must have a deterministic set of trajectories after some finite steps. Moreover, the set of trajectories have to coincide with that of master BN. However, since the slave BN is a probabilistic BN, the slave BN may have lots of possible trajectories, among which there may exists one possible trajectory coinciding with the trajectory of master BN. The main concern of synchronization in probability is that whether there exists one possible trajectory coinciding with the trajectory of master BN or not. The main difference between synchronization with probability one and synchronization in probability is that whether there exists one deterministic trajectory or one possible trajectory which coincides with the trajectory of master BN*.

Here, we still let 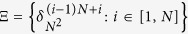 be the set of synchronized states about *z*(*t*). Let 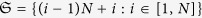 be the index set of Ξ and denote 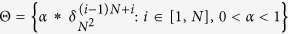. Based on Theorem 1, we have the following algebraic criterion for synchronization in probability.

**Theorem 3** *Consider the master-slave PBNs* (9). *System* (9) *can be synchronized in probability if and only if the following conditions hold:*

• *For* 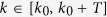,


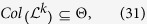


*where* 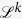 *is the matrix obtained from L*^*k*^ *by substituting zeros in the rows with index* 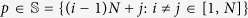;

•  


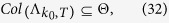


*where* 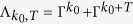, 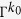 *and* 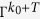 *are matrices obtained from* 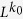 and 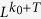 *by substituting zeros in the rows with index* 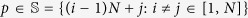.

**Proof**. (Sufficiency) Assuming that conditions (31) and (32) hold, we prove that under these conditions, the master BN can be synchronized in probability by the slave BN. It should be noted that for any initial state, the trajectory of master BN will enter into some cycle after *k*_0_ steps, i.e. 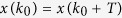. Moreover, the master BN is a deterministic BN. Thus, we only need to check whether the master BN can be synchronized in probability by slave BN at the limit states: 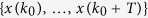. Suppose that 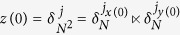, 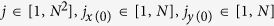. According to Eq. (27), we have 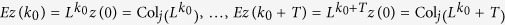. If condition (31) holds, it means that for each matrix 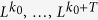, there is only one entry having a positive number in each column with index 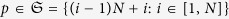. It implies that for any 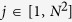 and after *k*_0_ steps, we have 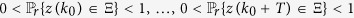. Thus, it implies that


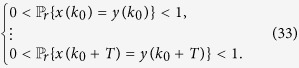


Moreover, it should be noted that the master BN is a deterministic BN. Hence, it will reach a cycle after *k*_0_ steps, i.e. 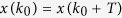. Condition (32) means that for each column of matrices 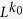 and 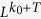, the index 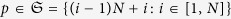 is the same. Then, if condition (32) holds, according to Eq. (27), we have


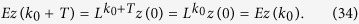


By this meaning, the slave BN can reach cycle coinciding with that of master BN with certain probability, i.e. 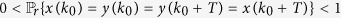. Thus, the master BN can be synchronized in probability by slave BN at the limit states: 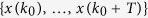. Hence, the master-slave PBNs (9) can be synchronized in probability.

(Necessity) If the master-slave PBNs (9) can be synchronized in probability, we prove that conditions (31) and (32) hold. Note that the master BN is a deterministic BN. Hence, it has exact trajectories. According to Proposition 1, we know that the trajectory will enter into certain cycle after *k*_0_ steps. Due to the fact that 0 < *α* < 1, there must also exist some positive number in the rows with index 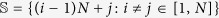. Thus, for 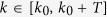, we must have 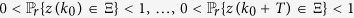, the probability can not be equal to 1. By this meaning, we have following equations:
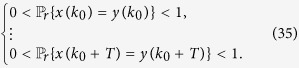


It implies that for any initial states *x*(0), *y*(0), we have


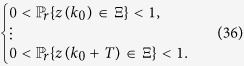


Thus, based on the equations of 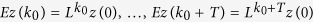, we derive that there exist some entries having positive number in each column with index 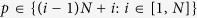 for each matrix 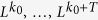. Moreover, since master BN is a deterministic BN which implies that each state 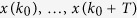 is deterministic, there is only one entry having positive number in each column with index 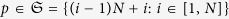 for each matrix 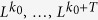. It implies that for 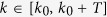, Col(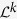) 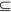 Θ, where 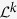 is the matrix obtained from *L*^*k*^ by substituting zeros in the rows with index 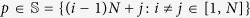.

Now, we prove condition (32) holds, provided the master-slave PBNs (9) can be synchronized in probability. Note that for any initial state *x*(0), we can always find *k*_0_ such that 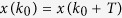. Thus, if the master-slave PBNs (9) can be synchronized in probability, it implies that 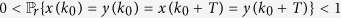. Thus, we have


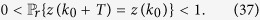


Let 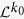 and 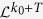 be the matrices obtained from 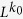 and 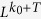 by substituting zeros in the rows with index 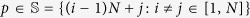. Since 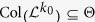 and 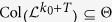, it implies that for each column of matrices 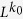 and 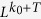, the index 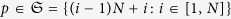 must be the same. By this meaning, we derive 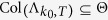, where 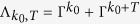, 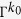 and 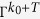 are matrices obtain from 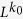 and 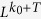 by substituting zeros in the rows with index 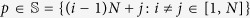. This completes the proof.

**Remark 10** *Due to the fact that the trajectory of master BN will enter into a cycle after k*_0_ *steps, we also only need to consider the time sequence k*_0_, *k*_0_ + 1, …, *k*_0_ + *T*. *Since the set* 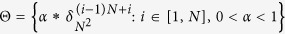, *if condition* (31) *holds, we can derive that* 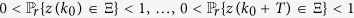 as 0 < *α* < 1. *Thus, we can conclude that at the time sequence k*_0_, *k*_0_ + 1, …, *k*_0_ + *T*, *the master-slave PBNs can reach synchronization in probability. Moreover, since* 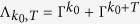, *condition* (32) *implies that the slave BN can generate one possible periodic trajectory with the same length as the trajectory of master BN. So, condition* (32) *guarantees that the master-slave PBNs can reach synchronization in probability after time k*_0_ + *T*.

**Theorem 4** *Consider the master-slave PBNs* (9). *The master-slave PBNs* (9) *can be synchronized in probability, if following two conditions hold:*

1. *there exists a positive number* 0 < *k* ≤ *k*_0_ + *T*, *such that Col*(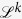) 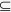 Θ, *where* 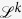 *is the matrix obtained from L*^*k*^ *by substituting zeros in the rows with index* 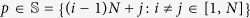;
2. *Col*_*j*_(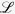) ∈ Θ, 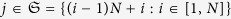, *where* 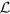 *is the matrix obtained from L by substituting zeros in the rows with index* 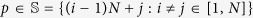.

**Proof**. Suppose that there exists a positive number 0 < *k* ≤ *k*_0_ + *T*, such that *Col*(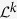) 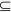 Θ, where 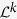 is the matrix obtained from *L*^*k*^ by substituting zeros in the rows with index 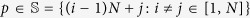. Then one can conclude that for each column of matrix *L*^*k*^, there is only one entry having positive number with certain index 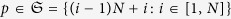. Then according to Eq. (27), for any initial state *z*(0), we have 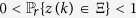, which implies 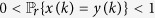. Suppose that there are *μ* possible states for *z*(*k*), denote


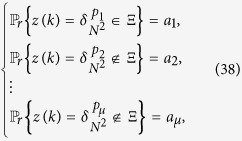


where 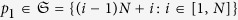, *p*_2_, …, *p*_*μ*_ ∈ 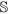, 0 < *a*_1_, *a*_2_, …, *a*_*μ*_. Considering the next step *t* = *k* + 1, we have


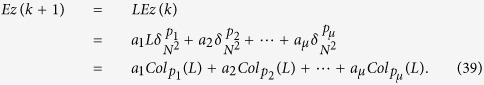


Since Col_*j*_(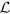) ∈ Θ, 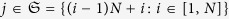 and 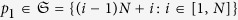, we can derive that 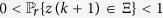, which implies that 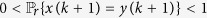. Thus, using mathematical iteration, we can obtain that 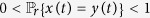, *t* ≥ *k*. Hence, the master-slave PBNs (9) can be synchronized in probability.

## Numerical Simulation

In this section, we present two numerical examples to demonstrate the applications of our main results.

**Example 6** *Let us consider the following two PBNs with* 2 *nodes coupled in the master-slave configuration:*


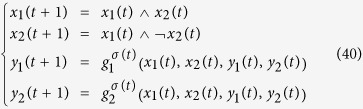


*where the switching signal is given by* 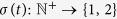 *and*


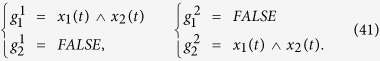


*Moreover, the probability for* 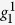, 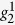, 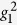 *and* 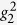 *are* 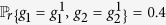, 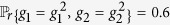. *The possible model index of matrix* 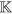 *is listed as follows:*


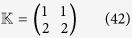


*Thus, there are* 2 *possible BNs for the slave BN to be chosen with some certain probability. One of the possible BN has the probability* 0.4*, while the other possible BN has the probability* 0.6.

*Our objective is to check whether these master-slave PBNs* (40) *can be synchronized with probability one or not. Denote* 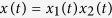 *and* 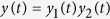. *By resorting to STP and Lemma* 1, *we can obtain its algebraic form of system* (40) *as follows:*


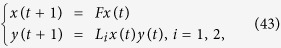


*where the probabilities of L*_*i*_ *being L*_1_ *and L*_2_ *are respectively* 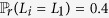 *and* 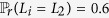, *and*


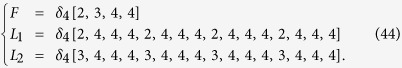


*Since the master-slave PBNs can be regarded as a whole system, we can obtain the following dynamics of whole system by letting* 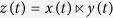: 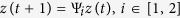, *where*


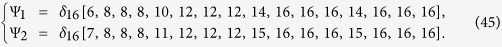


*Hence, we can obtain the overall expected value of z*(*t* + 1) *satisfies:*


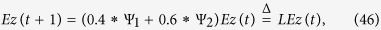


where


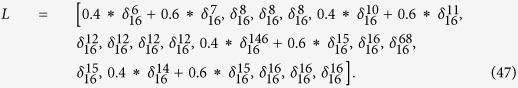


**Figure 3:**
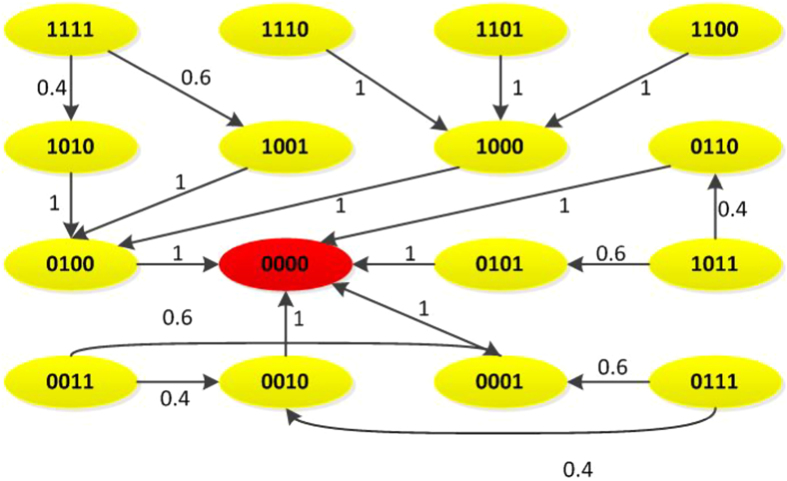
State transition digraph of system (40), where the synchronized state is filled with red colour.

*The state transition digraph of system* (40) *is shown in* Fig. 3.

*To apply Theorem* 1, *we firstly calculate the transient period of master BN k*_0_ *and the smallest positive number T* > 0 *satisfying* 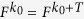. *According to Proposition* 1, *we can firstly obtain the transient period of master BN, i.e. k*_0_ = 3, *and the smallest positive number T* = 1 *satisfying F*^3^ = *F*^4^. *Then, according to Theorem* 1, *we can obtain that* 
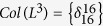
 *and L*^3^ = *L*^4^, *which implies that conditions* (25) *and* (26) *hold. Thus, this master-slave PBNs* (40) *can be synchronized with probability one. From* Fig. 3, *we observe that all the possible trajectories of system* (40) *starting from any initial state z*(0) ∈ 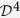 *will eventually enter into the synchronized state* (0, 0, 0, 0) *at the third time step, and it will never escape*.

**Example 7** *Now, we present another example to illustrate synchronization of master-slave PBNs in probability. Let us consider the following two PBNs with* 2 *nodes coupled in the master-slave configuration:*


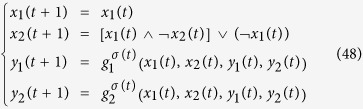


*where the switching signal is given by* 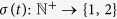 *and*


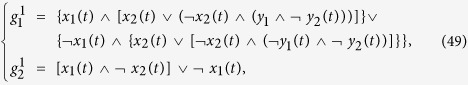



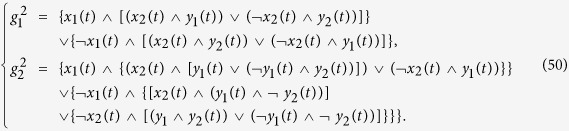


*Here, we let the probabilities be* 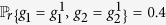 *and* 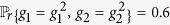. *Thus, we can obtain the following possible model index of matrix* 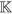, *which is listed as follows:*


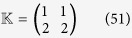


*Hence, the slave BN has* 2 *possible BNs to be chosen. One of the possible BN has the probability* 0.4, *while the other possible BN has the probability* 0.6.

*In order to check whether these two PBNs* (48) *can be synchronized in probability or not, we need to use Theorem* 3. *Denote* 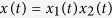 *and* 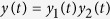. *By resorting to STP, we can obtain the following equivalent algebraic form of system* (48):


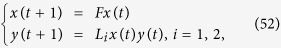


*where the probability of L*_*i*_ *being L*_1_ *and L*_2_ *is* 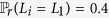 *and* 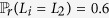, *and*


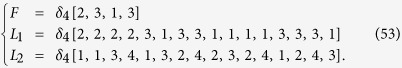


*Thus, we can obtain the overall expected value of z*(*t* + 1) *satisfies:*


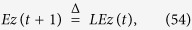


where


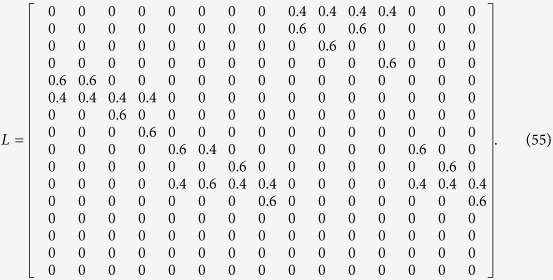


**Figure 4:**
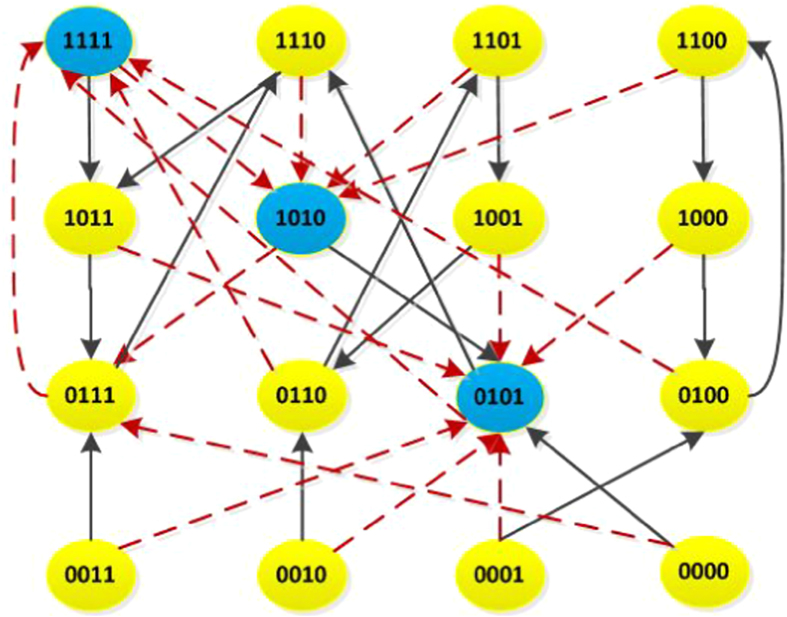
State transition digraph of system (48), where the synchronized states are filled with blue colour. The red dash line implies that state transfer to its next states with probability 0.4, while the black solid line implies that state transfer to its next states with probability 0.6.

*To better illustrate dynamic of the master-slave PBNs, the state transition digraph of system* (48) *is shown in* Fig. 4.

*Since the master BN is a deterministic BN, then we can firstly obtain the transient period of master BN k*_0_ *and the smallest positive number T* > 0 *satisfying* 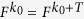, *which are k*_0_ = 1 *and T* = 3. *In order to check whether these two PBNs* (48) *can be synchronized in probability or not, we need to check whether conditions* (31) *and* (32) *hold or not. Firstly, we need to calculate matrices* 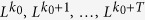, i.e. *L*, *L*^2^, *L*^3^, *L*^4^. *since* 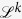 *is the matrix obtained from L*^*k*^ *by substituting zeros in the rows with index* 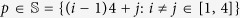, *it is easy to check that for k* = 1, 2, 3, 4, *Col*(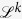) 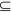 Θ. *Secondly, since* Λ_1,3_ = Γ + Γ^4^, Γ *and* Γ^4^ *are matrices obtained from L and L*^4^ *by substituting zeros in the rows with index* 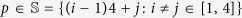, *one can conclude that Col*(Λ_1,3_) 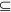 Θ. *Thus, according to Theorem* 4, *this master-slave PBNs* (48) *can be synchronized in probability*.

**Figure 5:**
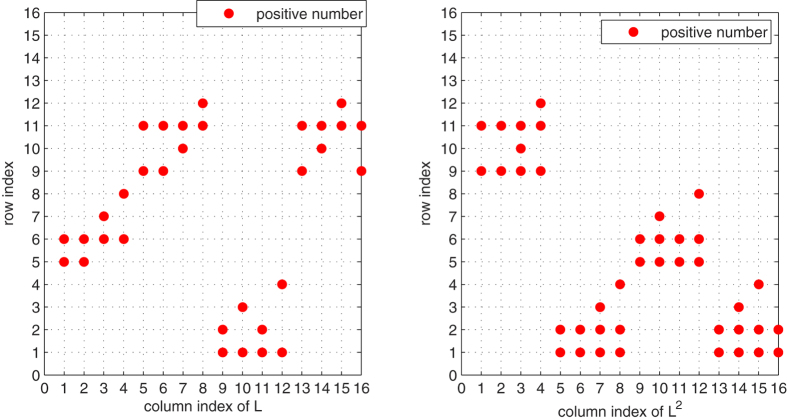
The row index for each column of matrices *L* and *L*^2^ in system (48). Each point corresponds to the row index, in which has a positive number in corresponding matrices *L* and *L*^2^.

**Figure 6:**
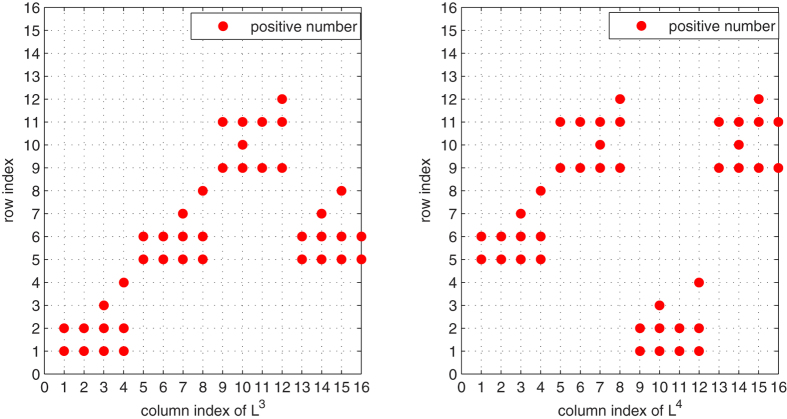
The row index for each column of matrices *L*^3^ and *L*^4^ in system (48). Each point corresponds to the row index, in which has a positive number in corresponding matrices *L*^3^ and *L*^4^.

**Figure 7:**
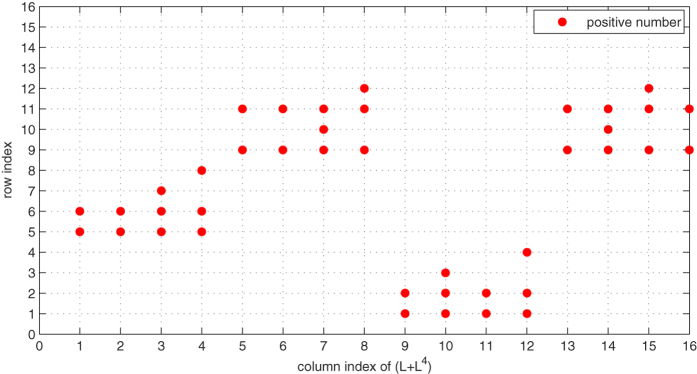
The row index for each column of matrices *L* + *L*^4^ in system (48). Each point corresponds to the row index, in which has a positive number in corresponding matrices *L* + *L*^4^.

*In* Figs 5 and 6, *the row index having positive number of each column L*^*i*^, *i* = 1, 2, 3, 4 *are plotted. And* Fig. 7 *plots the row index having positive number of each column of L* + *L*^4^. *From* Figs 5 and 6, *we can draw a conclusion that for each column of matrices L*, *L*^2^, *L*^3^, *L*^4^ *and* (*L* + *L*^4^), *there is only one index* 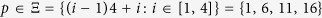 *having a positive number. Thus, it implies that conditions* (31) *and* (32) *hold in the same way, which well illustrate our main results*.

## Conclusions

In this paper, both synchronization of master-slave PBNs with probability one and synchronization in probability have been investigated. One restriction in this paper is that master BN is a deterministic BN, while slave BN is a probabilistic BN. Slave BN is determined by a series of possible logical functions with certain probability at each time point. The definitions of synchronization with probability one and synchronization in probability are firstly presented in this paper. Due to the fact that the master BN is a deterministic BN while the slave BN is a probabilistic BN, this paper considers two different cases: synchronization with probability one and synchronization in probability. The main concern of synchronization in probability is that whether there exists one possible trajectory coinciding with the trajectory of master BN or not. The main difference between synchronization with probability one and synchronization in probability is that whether there exists one deterministic trajectory or one possible trajectory which coincides with the trajectory of master BN. Based on STP and its equivalent algebraic form, several necessary and sufficient conditions for two types of synchronization are derived. According to obtained necessary and sufficient conditions, we derive some effective conditions to judge whether some given master-slave PBNs can be synchronized with probability one or not. And then, some effective conditions are also obtained to judge whether some given master-slave PBNs can be synchronized in probability or not. Moreover, the main results are well illustrated by numerical examples.

Unfortunately, determining whether the master-slave PBN can be synchronized or not is still NP-hard. Some interesting and meaningful topics that deserve further research include the following: to investigate synchronization problem with different (or time-varying) delays, to investigate the feedback controller based on switching signals, and so on.

## Additional Information

**How to cite this article**: Lu, J. *et al.* Synchronization Analysis of Master-Slave Probabilistic Boolean Networks. *Sci. Rep.* **5**, 13437; doi: 10.1038/srep13437 (2015).

## Figures and Tables

**Table 1:** Truth table.

*a*	*b*	¬*a*	*a * 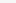 * b*	(¬*a *) 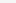 * b*
1	1	0	1	1
1	0	0	1	0
0	1	1	1	1
0	0	1	0	1
